# Neuroanatomical substrates of perivascular space index: a morphometric and tractography study

**DOI:** 10.3389/fpsyt.2026.1848053

**Published:** 2026-06-19

**Authors:** Xuyang Wang, Qin Zhang, Peitao Su, Wenjing Lu, Guoqiang Chen, Ping Cui, Mao Sheng, Shiwu Yin

**Affiliations:** 1 Department of Radiology, The Second People’s Hospital of Hefei, Hefei Hospital Affiliated to Anhui Medical University, Hefei, Anhui, China; 2 Affiliations 1 Department of Interventional Vascular Medicine, Hefei Hospital Affiliated to Anhui Medical University, The Second People’s Hospital of Hefei, Hefei, Anhui, China; 3 The Fifth Clinical College of Medicine, Anhui Medical University, Hefei, Anhui, China; 4 Wannan Medical College, Wuhu, Anhui, China

**Keywords:** DTI-ALPS index, glymphatic system, surface-based morphometry, tractography, white matter microstructure

## Abstract

**Background and objectives:**

The diffusion tensor image analysis along the perivascular space (DTI-ALPS) index is an important imaging biomarker for assessing glymphatic system function. However, the cortical and white matter metrics associated with changes in the DTI-ALPS index have not been fully elucidated, and the relationship between these two types of metrics remains controversial.

**Methods:**

We computed the index in 815 healthy participants from the AOMIC dataset. Surface-based morphometry (SBM) and correlational tractography were employed to identify cortical morphometric features and white matter tracts that were correlated with the DTI-ALPS index. Tract-to-region connectome analysis was then performed to identify significant tracts, and correlations between tract microstructural integrity and connected cortical morphometry were analyzed.

**Results:**

The SBM results revealed positive correlations between the DTI-ALPS index and sulcal depth in the precentral, parsopercularis, postcentral, and supramarginal gyri as well as with local gyrification in the superior frontal and lateral orbitofrontal cortices. Tractography showed that the DTI-ALPS index positively correlated with fractional anisotropy (FA) and negatively with mean diffusivity (MD) in the corpus callosum, bilateral fornices, and corticospinal tracts. These tracts exhibited structural connectivity with the aforementioned cortical regions, and MD in several connected tracts positively correlated with regional sulcal depth.

**Discussion:**

This study identified cortical and white matter correlates of DTI-ALPS variability in a healthy young-adult cohort. These findings provide novel insights into mechanisms underlying DTI-ALPS index decline in aging and neurological disorders.

## Introduction

In the glymphatic system, cerebrospinal fluid (CSF) produced by the choroid plexus enters the brain along perivascular spaces (PVS) surrounding pial and perforating arteries, penetrates the brain parenchyma via aquaporin-4 (AQP4) water channels located on astrocytic end-feet, mixes with interstitial fluid (ISF), and subsequently drains into perivenous and perineuronal spaces, thereby facilitating the clearance of neurotoxic proteins and metabolic waste (1–3). However, CSF dynamics exhibit considerable regional heterogeneity and do not strictly conform to the anatomical distribution of perivascular networks (4, 5). White matter tracts serve as important alternative conduits for intracerebral fluid transport (3, 6). Moreover, CSF flow velocity is intimately associated with cortical morphology, with gyral folding accelerating CSF dispersion (7). Thus, cerebral morphological architecture provides the structural foundation for glymphatic function. Nevertheless, the specific white matter components contributing to glymphatic clearance remain incompletely elucidated, and the influence of cortical morphology on glymphatic function remains poorly characterized.

The diffusion tensor image analysis along the perivascular space (DTI-ALPS) index represents a noninvasive neuroimaging biomarker for assessing glymphatic function (8). This metric has demonstrated robust test–retest reproducibility in prior validation studies (9, 10) and has been increasingly applied in research on stroke, Alzheimer’s disease, Parkinson’s disease, sleep disorders, and type 2 diabetes (11–14). Postmortem investigations by Li and colleagues revealed that the DTI-ALPS index reflects fluid dynamics within perivascular spaces modulated by adjacent white matter architecture, with particularly strong associations observed for specific fiber tracts (15). Nevertheless, the precise white matter components governing DTI-ALPS index variability remain undefined in large-scale cohorts of healthy individuals. Although voxel-based morphometry has been employed to examine relationships between the DTI-ALPS index and regional gray matter volume in middle-aged and older adults (16), this approach possesses inherent methodological constraints that preclude comprehensive evaluation of how cortical folding geometry influences glymphatic efficiency.

This study aimed to investigate associations between the DTI-ALPS index and gray and white matter morphological features using surface-based morphometry (SBM) and correlational tractography. SBM was employed to quantify cortical morphometric parameters, including sulcal depth and gyrification index (17). Correlational tractography—a novel diffusion MRI (dMRI) analytical framework—offers superior sensitivity compared with conventional voxel- or tract-based approaches and enables the identification of white matter tracts correlated with the DTI-ALPS index through permutation testing (18). To mitigate potential bias arising from reliance on fractional anisotropy alone, mean diffusivity (MD) was incorporated as a local connectome fingerprint within the correlational tractography. As a rotationally invariant diffusion tensor metric, MD provides a robust indicator of tissue cellular density and has emerged as a sensitive marker of neuroplasticity; concurrent increases in anisotropy with reductions in MD offer compelling evidence of enhanced axonal density and microstructural integrity (19). In addition, we performed a tract-to-region connectome analysis (20) to identify the white matter components that connect correlational white matter tracts to cortical regions and to explore the potential associations between them.

Based on accumulating evidence, we hypothesized that (1) glymphatic function, as indexed by the DTI-ALPS metric, is closely associated with the microstructural integrity of white matter tracts and (2) greater cortical thickness, sulcal depth, and gyrification are associated with elevated DTI-ALPS index values. Validating these hypotheses in a large cohort of healthy young adults is critical to elucidate the neuroanatomical substrates of intracerebral fluid clearance and the pathophysiological mechanisms underlying its age- and disease-related decline.

## Methods

### Participants

**Figure 1:**
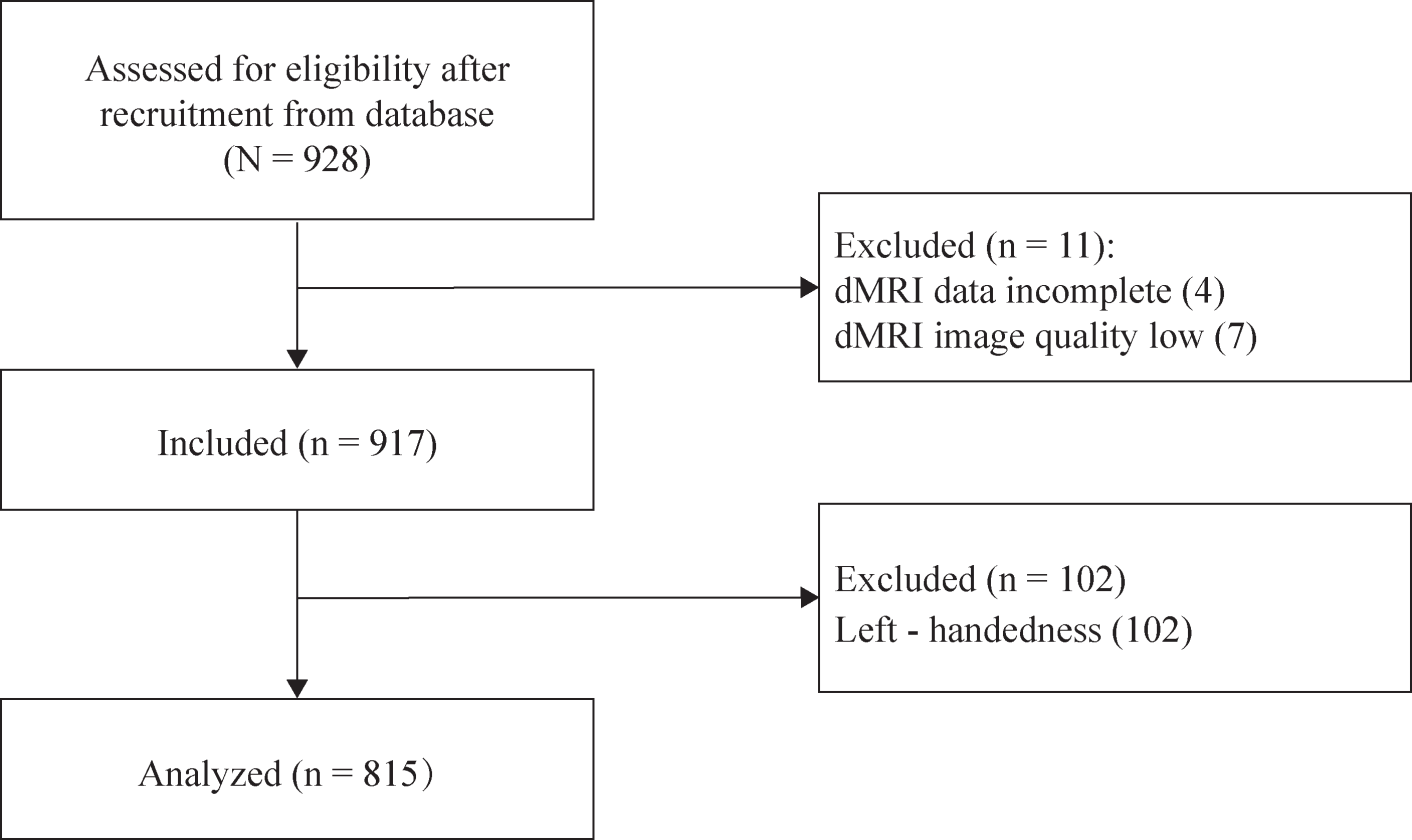
Participant flow diagram.

This study utilized dMRI and structural MRI data from the Amsterdam Open MRI Collection ID1000 (AOMIC ID1000) dataset (21), a publicly available multimodal neuroimaging repository comprising 928 healthy participants with comprehensive demographic information. The dataset is available at https://openneuro.org/datasets/ds003097. All MRI acquisitions were performed on a single Philips 3T scanner (Philips Healthcare, Best, The Netherlands). Participants were excluded for missing data, suboptimal image quality, or left-handedness to minimize potential confounding effects of cerebral lateralization. The participant selection flowchart is presented in **Figure 1**. The final analytical sample consisted of 815 participants (425 women and 390 men). Demographic characteristics are summarized in **Table 1**.

**Table 1:** Demographic, cognitive, and psychological characteristics of the participants.

Characteristics	Participants
Sex	
Male	390 (47.9%)
Female	425 (52.1%)
Education level	
Low	79 (9.7%)
Medium	354 (43.4%)
High	382 (46.9%)
Age	22.8 (21.2, 24.2)
BMI	23.0 (21.0, 26.0)
Background_SES	4.0 (3.0, 5.0)
IST_fluid	109.0 (90.0, 126.0)
IST_memory	55.0 (49.0, 61.0)
IST_crystallized	39.0 (32.0, 46.0)
IST_intelligence_total	204.0 (172.2, 230.0)
BAS_drive	11.0 (9.0, 12.0)
BAS_fun	11.0 (10.0, 13.0)
BAS_reward	16.0 (15.0, 17.0)
BIS	20.0 (17.0, 22.0)
NEO_N	32.0 (28.0, 36.0)
NEO_E	42.0 (38.0, 46.0)
NEO_O	41.0 (36.0, 45.0)
NEO_A	40.0 (36.0, 44.0)
NEO_C	38.0 (34.0, 42.0)
STAI_T	38.0 (31.0, 44.0)

BAS, behavioral activation system; BIS, behavioral inhibition system; IST, Intelligence Structure Test; NEO, NEO Five-Factor Inventory; STAI, State-Trait Anxiety Inventory.

### DTI-ALPS index calculation

Preprocessed dMRI data in NIfTI format—processed through a customized pipeline integrating MRtrix3 and FSL tools within the AOMIC ID1000 dataset—were converted to SRC format using DSI Studio (https://dsi-studio.labsolver.org). Images with suboptimal quality or inconsistent slice numbers were excluded following automated quality control. Data reconstruction was performed in MNI152 standard space using q-space diffeomorphic reconstruction, yielding FIB files from which diffusion maps along the x-, y-, and z-axis, respectively, and fiber orientation maps were extracted.

**Figure 2:**
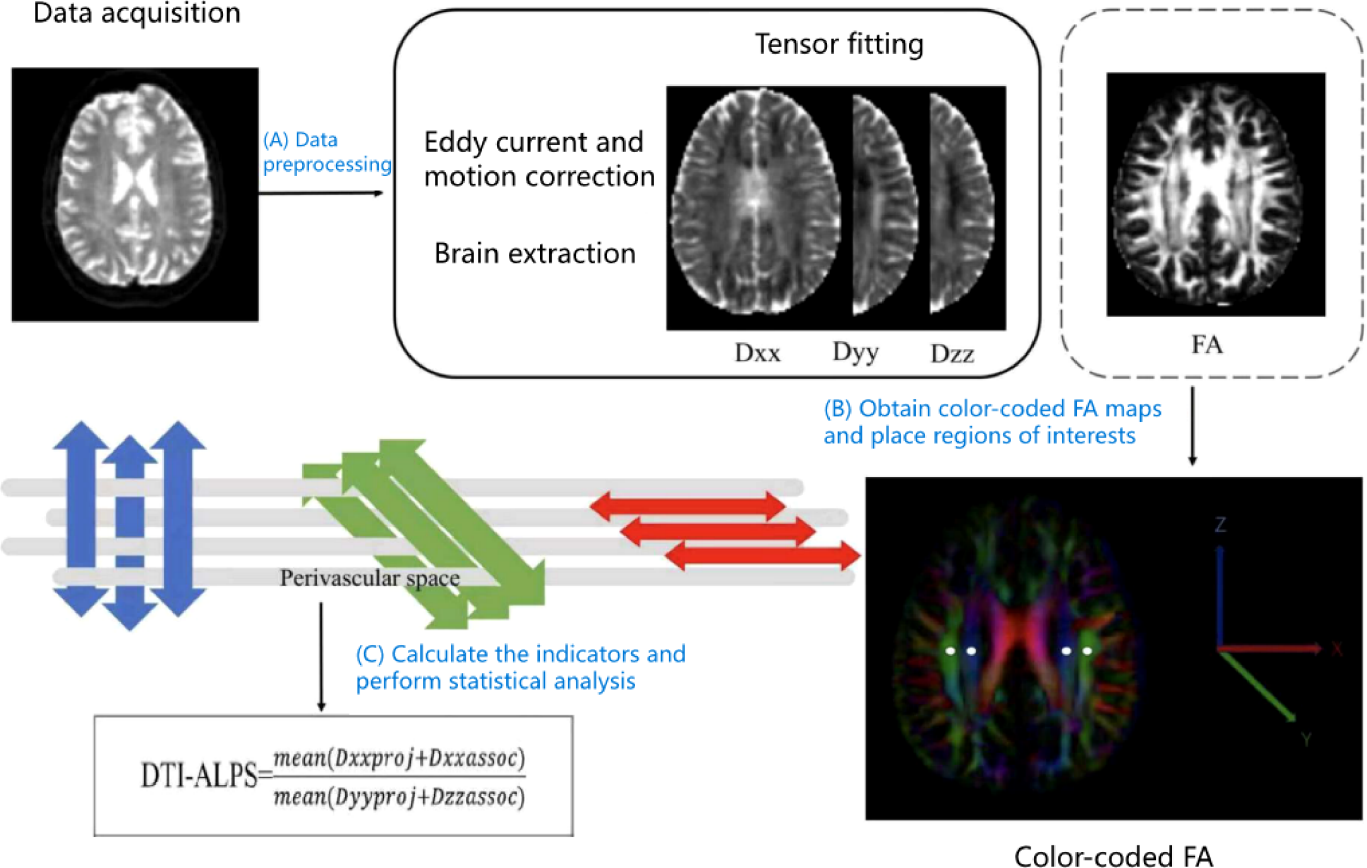
Schematic illustration of the DTI-ALPS method. **(a)** DTI data preprocessing pipeline. **(b)** Generation of color-coded fractional anisotropy (FA) maps with region-of-interest placement in standard space to quantify diffusivity along projection and association fibers. In the color FA map, blue, green, and red voxels denote the predominant orientations of projection fibers (superior-inferior, z-axis), association fibers (anterior-posterior, y-axis), and commissural fibers (left-right, x-axis), respectively. Gray cylinders represent perivascular spaces surrounding medullary veins; arrows indicate the principal diffusion directions of projection (blue), association (green), and commissural (red) fiber tracts. **(c)** Computation of the DTI-ALPS index for subsequent statistical analysis. FA, fractional anisotropy; DTI-ALPS, diffusion tensor image analysis along the perivascular space.

As illustrated in **Figure 2**, spherical ROIs (6 mm in diameter) were placed at coordinates from a previous multicenter study (10), ensuring placement within projection and association fiber trajectories: left hemisphere projection fibers (−25, −47, 40) and association fibers (−19, −47, 40); right hemisphere projection fibers (25, −47, 40) and association fibers (19, −47, 40). Mean diffusivities along the x-, y-, and z axis, respectively, were extracted for each ROI (Dx_proj, Dx_assoc, Dy_proj, Dy_assoc, Dz_proj, and Dz_assoc). The DTI-ALPS index for each hemisphere was calculated as follows: DTI-ALPS index = mean(Dx_proj, Dx_assoc)/mean(Dy_proj, Dz_assoc). The final DTI-ALPS index was defined as the average of left and right hemisphere values.

### Cortical morphometric analysis

Cortical segmentation and morphometric processing were performed using the Computational Anatomy Toolbox 12.7 (CAT12; http://dbm.neuro.uni-jena.de/cat/) implemented within Statistical Parametric Mapping 12 (SPM12; https://www.fil.ion.ucl.ac.uk/spm/). This pipeline incorporated spherical-harmonics-based topology correction for tissue segmentation and spherical registration for spatial normalization to standard space. Cortical morphometric parameters—including cortical thickness, sulcal depth, and gyrification—were extracted based on the Desikan–Killiany (DK) atlas. All structural MRI scans achieved a preprocessing quality rating of B− or higher according to CAT12’s internal quality control metrics, with mean weighted images meeting stringent inclusion criteria.

Linear regression models were constructed using SPM12, with the DTI-ALPS index as the study variable and sex, age, and education level included as covariates. Brain-region-based morphological analysis was performed using CAT12. After false discovery rate (FDR) correction, *p <*0.05 was considered statistically significant.

### Correlational tractography analysis

This study constructed a connectome database comprising 815 participants based on local connectome matrices reconstructed from individual dMRI datasets. In dMRI, higher white matter integrity and greater fiber density enhance tissue restriction of water diffusion, manifesting as increased fractional anisotropy (FA) and decreased MD. Building upon a previously established methodology (22), we extracted FA and MD values as local connectome fingerprints to quantify structural connectivity. Deterministic fiber tracking was applied to select and trace local connections exceeding a *t*-statistic threshold of 2.5. A minimum fiber length threshold of 20 voxels was imposed to identify biologically plausible tracts. The DTI-ALPS index served as the primary variable of interest, with sex, age, and education level included as covariates; cerebellar regions were excluded from all analyses. Spurious connections were eliminated through four iterative rounds of topological refinement. To assess statistical significance 4,000 bootstrap permutations with randomization were performed to estimate the null distribution of tract lengths, and multiple comparisons were corrected using the FDR procedure. The resulting tracts were automatically segmented into distinct fiber bundles using DSI Studio. Cortical regions were parcellated using the FreeSurfer DK atlas and reconstructed in ICBM152 standard space. Using the tract-to-region connectome analysis method, we measured the probability that specific white matter tracts innervate particular cortical regions. A probability greater than 0 was considered indicative of an anatomical connection. Fiber tracts demonstrating such anatomical connectivity with cortical regions were considered to provide structural support and were retained for subsequent analyses.

### Correlation analysis

Partial correlation analyses were performed to examine the relationships between cortical morphometric measures and diffusion metrics (FA or MD) of structurally connected white matter tracts, with sex, age, and education level entered as covariates. Only tract–region pairs with documented anatomical connections were included. To account for the seven comparisons conducted, a Bonferroni correction was applied by multiplying the raw *p*-values by 7, with adjusted *p <*0.05 considered statistically significant.

## Results

**Figure 3:**
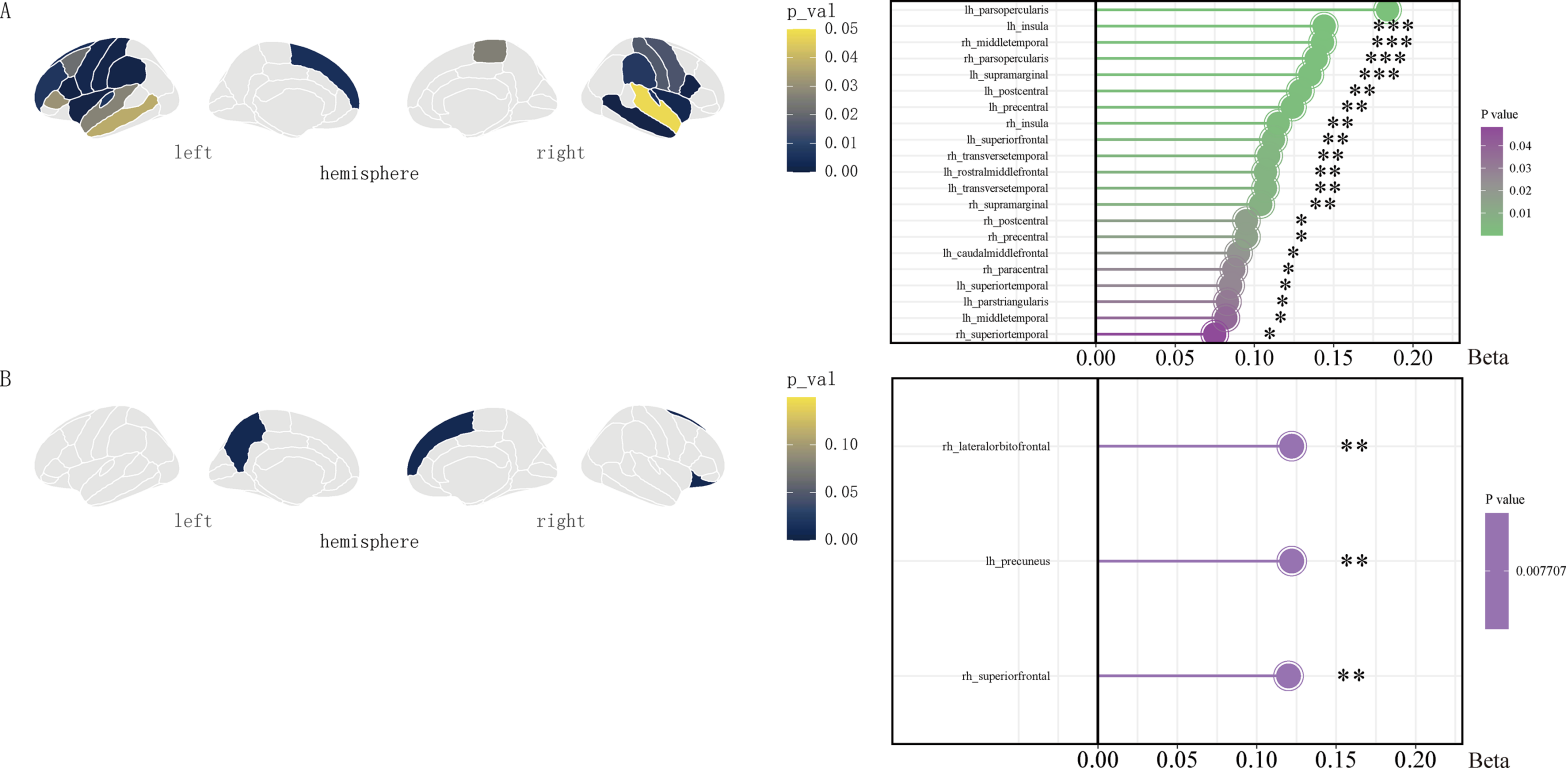
Associations between the diffusion tensor image analysis along the perivascular space (DTI-ALPS) index and cortical morphometric measures in regions of interest defined by the Desikan–Killiany atlas. **(A)** Positive correlations were observed between DTI-ALPS index and sulcal depth in the left postcentral gyrus, precentral gyrus, and superior frontal gyrus as well as in the right postcentral gyrus, precentral gyrus, supramarginal gyrus, and insula. **(B)** Positive correlations were identified between DTI-ALPS index and gyrification index in the left precuneus and the right superior frontal gyrus and lateral orbitofrontal cortex. *p < 0.05, **p < 0.01, ***p < 0.001

Cortical morphometric analysis demonstrated that sulcal depth in the bilateral precentral gyrus, postcentral gyrus, supramarginal gyrus, insula, pars opercularis, transverse temporal gyrus, superior temporal gyrus, middle temporal gyrus, left superior frontal gyrus, rostral middle frontal gyrus, caudal middle frontal gyrus, and pars triangularis, as well as in the right paracentral lobule, was positively correlated with the DTI-ALPS index across the whole cortex (FDR-corrected *p* < 0.05; **Figure 3A**). Additionally, local gyrification in the right superior frontal gyrus and lateral orbitofrontal cortex, as well as in the left precuneus, showed positive correlations with the DTI-ALPS index (FDR-corrected *p* < 0.05; **Figure 3B**). No correlation was observed between whole-cortex thickness and the DTI-ALPS index. Detailed results are provided in **Table 2**.

**Table 2:** Brain regions where the morphological structure correlates with the DTI-ALPS index.

Atlas-based ROI	Beta	P-value	*B*	SE
Depth				
lh_parsopercularis	0.184	0.000003	1.67	0.31
lh_insula	0.144	0.000244	1.425	0.326
lh_supramarginal	0.135	0.000786	0.802	0.206
lh_postcentral	0.129	0.001308	0.562	0.151
lh_precentral	0.124	0.00169	0.552	0.153
lh_superiorfrontal	0.112	0.004798	0.322	0.099
lh_transversetemporal	0.107	0.006131	1.256	0.403
lh_rostralmiddlefrontal	0.107	0.006271	0.418	0.135
lh_caudalmiddlefrontal	0.09	0.021566	0.534	0.206
lh_superiortemporal	0.085	0.027069	0.596	0.241
lh_parstriangularis	0.083	0.031223	0.544	0.227
lh_middletemporal	0.082	0.037568	0.339	0.147
rh_middletemporal	0.143	0.00052	0.51	0.124
rh_parsopercularis	0.139	0.000675	1.364	0.343
rh_insula	0.115	0.002256	1.15	0.329
rh_transversetemporal	0.109	0.005383	1.363	0.428
rh_supramarginal	0.104	0.007415	0.688	0.228
rh_precentral	0.095	0.015052	0.397	0.144
rh_postcentral	0.095	0.016376	0.442	0.163
rh_paracentral	0.087	0.026025	0.443	0.177
rh_superiortemporal	0.075	0.04822	0.501	0.228
Gyrification				
lh_precuneus	0.122	0.007707	0.761	0.222
rh_superiorfrontal	0.12	0.007707	0.676	0.191
rh_lateralorbitofrontal	0.122	0.007707	0.879	0.251

All *P*-values were FDR-corrected using the Benjamini–Hochberg method.

**Figure 4:**
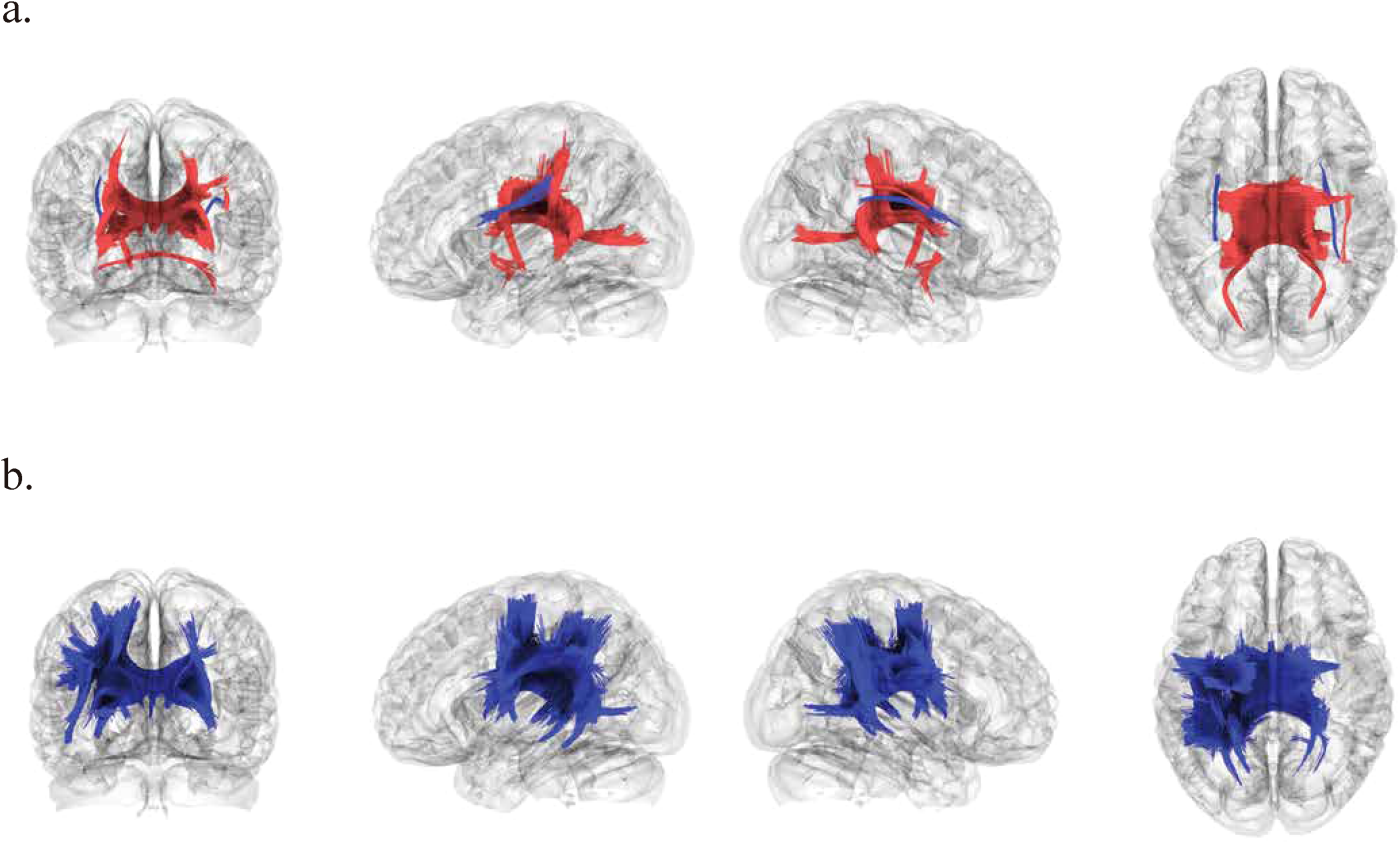
Schematic illustration of the relationship between the diffusion tensor image analysis along the perivascular space (DTI-ALPS) index and white matter tracts using fractional anisotropy (FA) **(a)** and mean diffusivity (MD) **(b)** as local connectome fingerprints. White matter tracts showing significant correlations with the DTI-ALPS index were reconstructed using DSI Studio. Periventricular projection and association fibers bilaterally exhibited correlations with the DTI-ALPS index: red streamlines indicate positive correlations (higher DTI-ALPS index associated with higher dMRI metrics), whereas blue streamlines indicate negative correlations (higher DTI-ALPS index associated with lower dMRI metrics).

After controlling for age, sex, and education level, we observed that the DTI-ALPS index was positively correlated with FA in fibers such as the corpus callosum, bilateral fornices, corticospinal tracts, and superior thalamic radiations in healthy young adults (FDR-corrected *p* < 0.05; **Figure 4a**; **Supplementary Table 1**). In addition, a limited number of negative correlations were found in the right arcuate fasciculus, left arcuate fasciculus, and right superior longitudinal fasciculus (**Supplementary Table 2**). When MD was used as the local connectome fingerprint metric, all identified white matter tracts demonstrated negative correlations with the DTI-ALPS index (FDR-corrected *p* < 0.05; **Figure 4b**; **Supplementary Table 3**). Detailed tract-specific correlation coefficients and morphometric data are provided in the **Supplementary Materials**.

**Figure 5:**
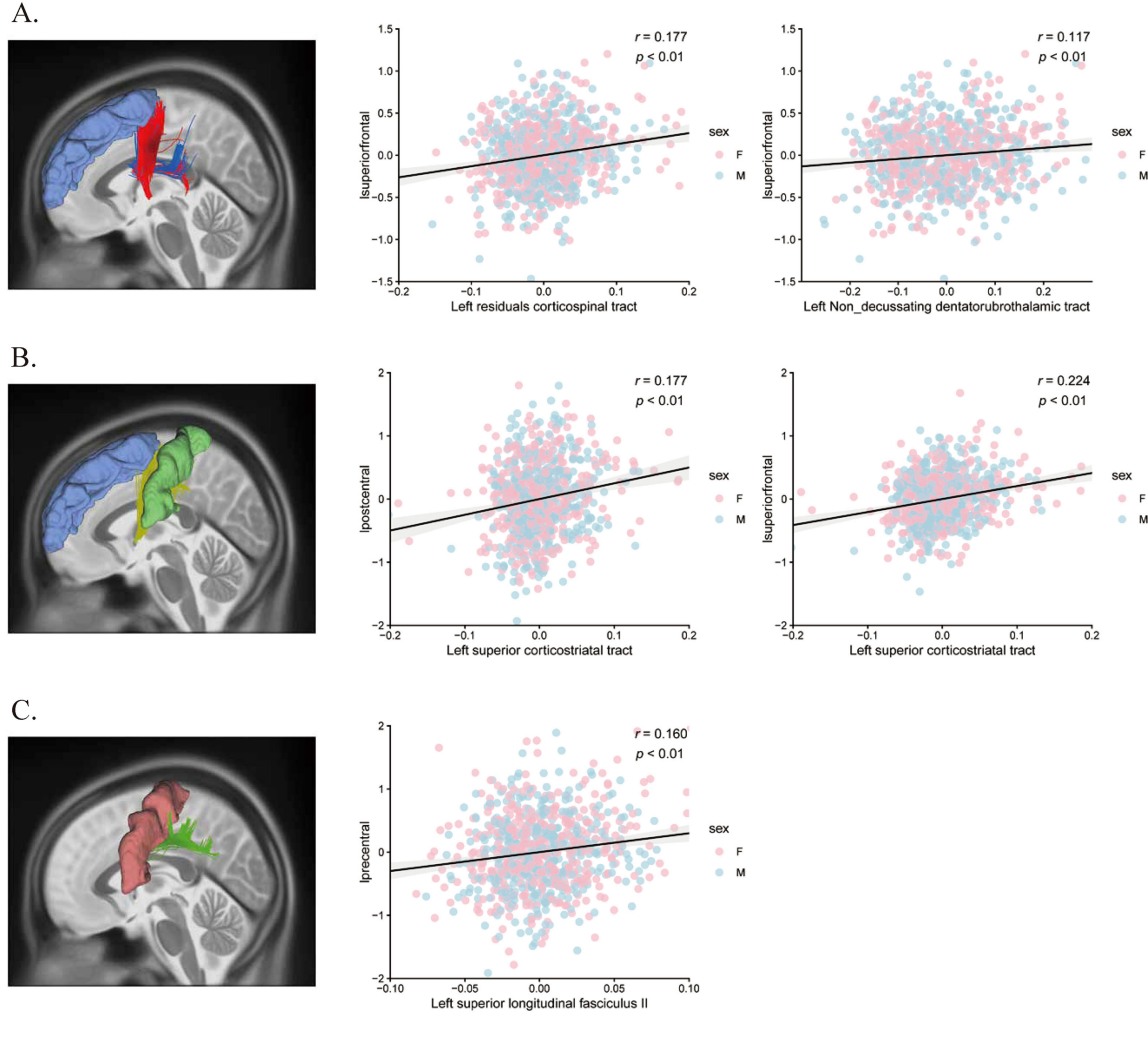
Partial correlation scatter plots between diffusion MRI (dMRI) metrics of white matter tracts and cortical morphometric measures, with sex, age, and education level controlled as covariates. **(A)** The mean diffusivity (MD) of the left corticospinal tract (red) and non-decussating dentatorubrothalamic tract (blue) showed positive correlations with sulcal depth in the superior frontal gyrus (blue). **(B)** The MD of the superior component of the left corticostriatal tract (yellow) was positively correlated with sulcal depth in the postcentral gyrus (green) and superior frontal gyrus. **(C)** The MD of the left superior longitudinal fasciculus (green) exhibited a positive correlation with sulcal depth in the precentral gyrus (red).

White matter tracts and cortical regions significantly correlated with the DTI-ALPS index were extracted using DSI Studio. A total of seven tract–region pairs were selected for correlation analysis based on anatomical connectivity: left postcentral gyrus with the superior component of the left corticostriatal tract, left precentral gyrus with the left arcuate fasciculus, left precentral gyrus with the left superior longitudinal fasciculus, left superior frontal gyrus with the left corticospinal tract, left superior frontal gyrus with the left non-decussating dentatorubrothalamic tract, left superior frontal gyrus with the left medial lemniscus tract, and left corticostriatal tract with the superior frontal gyrus. As illustrated in **Figure 5**, mean sulcal depth in the superior frontal gyrus showed positive correlations with MD values of the left corticospinal tract (*r* = 0.177, *p* = 2.46e-06 after Bonferroni correction) and the left non-decussating dentatorubrothalamic tract (*r* = 0.117, *p* = 5.85e-03 after Bonferroni correction). The MD of the superior component of the left corticostriatal tract correlated positively with mean sulcal depth in both the postcentral gyrus (*r* = 0.177, *p* = 2.57e-06 after Bonferroni correction) and the superior frontal gyrus (*r* = 0.224, *p* = 7.42e-10 after Bonferroni correction). Additionally, the MD of segment 2 of the left superior longitudinal fasciculus was positively associated with mean sulcal depth in the precentral gyrus (*r* = 0.160, *p* = 3.26e-04 after Bonferroni correction).

## Discussion

This study identified cortical and white matter fiber indicators associated with variations in the DTI-ALPS index in a large sample of healthy young adults. Individuals with a higher DTI-ALPS index tended to exhibit higher white matter integrity of the projection and association fibers in regions along the trajectory of the medullary veins, as well as deeper and more complex sulcal patterns in certain cortical areas. Furthermore, we observed an association between the integrity of certain fibers that influence the DTI-ALPS index and sulcal depth.

Previous postmortem investigations by Li et al. demonstrated the presence of the DTI-ALPS index in human brains and its close association with white matter fiber architecture, suggesting that the DTI-ALPS index may reflect the combined influence of perivascular fluid dynamics and white matter microstructure (15). White matter tracts serve as critical conduits for CSF movement and, during neurodevelopment, exert mechanical traction forces that shape cortical folding—forming sulci and gyri—which shortens anatomical distances between functional regions and provides a structural substrate for efficient neural integration. Notably, arteries coursing along sulcal surfaces possess perivascular spaces that constitute one of the primary routes for CSF influx into the brain parenchyma (7). Consistent with these findings, our study in healthy young adults identified robust correlations between white matter microstructural integrity and the DTI-ALPS index. In conditions such as Alzheimer’s disease and type 2 diabetes, a diminished DTI-ALPS index has been predominantly attributed to impaired CSF clearance secondary to neurotoxic protein deposition within perivascular spaces (23–25). Similarly, studies of mood disorders have reported reduced AQP4 expression and diminished glial-specific markers in the hippocampus and cingulate cortex (26, 27). However, these pathologies frequently co-occur with widespread white matter damage. We propose that structural compromise of white matter tracts—which function as essential channels for CSF transport within the parenchyma—may also contribute to disrupted glymphatic flow, thereby manifesting as decreased DTI-ALPS index on neuroimaging.

Tract-based correlation analysis revealed significant associations between the DTI-ALPS index and the microstructural properties of white matter tracts, including the corpus callosum, fornix, and corticospinal tracts. These fiber bundles predominantly serve as major interhemispheric pathways and are anatomically distributed within periventricular regions adjacent to medullary veins. The corpus callosum, the largest white matter tract in the human brain, and the fornix, a critical component of the hippocampal circuitry, both play central roles in bilateral hemispheric integration. Emerging evidence indicates that neural activity enhances glymphatic fluid dynamics (28), which may partially explain the influence of the corpus callosum and fornix on perivascular clearance efficiency. Given that corpus callosal integrity is frequently compromised across multiple neurological disorders (29–32), its structural deterioration may represent a key substrate underlying impaired glymphatic function. Furthermore, the subcallosal region is anatomically connected to the medial and intermediate olfactory striae of the olfactory nerve, a pathway implicated as a primary route for cerebrospinal fluid efflux from perineuronal spaces to cervical lymph nodes (33, 34). Consequently, interstitial compartments within white matter tracts may not only constitute intrinsic conduits for intraparenchymal cerebrospinal fluid movement but also provide structural foundations for extraparenchymal clearance routes such as perineuronal spaces. Nevertheless, the precise mechanistic interplay between these pathways warrants further investigation to achieve a comprehensive understanding of glymphatic–structural interactions. Notably, this study found an unexplained negative correlation between FA and the DTI-ALPS index in the right arcuate fasciculus, left arcuate fasciculus, and right superior longitudinal fasciculus, whereas no similar issue was observed in correlational tractography using MD metrics. It is possible that tracts exhibiting negative associations are those with crossing fibers that artificially lower FA, independently of true microstructural damage.

SBM analysis revealed significant positive correlations between the DTI-ALPS index and both sulcal depth and local gyrification in specific cortical regions, consistent with our initial hypothesis. Prior evidence indicates that CSF flow velocity is higher within sulci than gyri, and sulcal regions harbor a greater density of large-caliber arteries. Cortical folding in humans and most mammals arises from mechanical traction exerted by underlying white matter bundles during development; consequently, pial arteries predominantly course within sulci, and their perivascular spaces serve as critical conduits for CSF influx into deep brain parenchyma (35). Deeper sulci and more complex gyrification may therefore facilitate greater CSF inflow, necessitating enhanced water diffusivity within perivenous spaces to maintain overall fluid homeostasis. Clinically, neuroinflammatory edema and hydrocephalus secondary to CSF circulation impairment are frequently associated with sulcal effacement. We propose that, in addition to reduced arterial pulsatility due to arteriosclerosis (36), sulcal shallowing itself may represent a structural contributor to decreased DTI-ALPS index values. Notably, although frontotemporal dementia has been associated with correlations between cortical thickness and the DTI-ALPS index (37), our analysis in healthy young adults identified no significant regional associations between cortical thickness and glymphatic function. This suggests that, in neurologically intact young populations, sulcal morphology shows a stronger association with the DTI-ALPS index than cortical thickness does.

Surface-based morphometry revealed positive correlations between the DTI-ALPS index and both sulcal depth and gyrification index in cortical regions, including the postcentral and superior frontal gyri. These regions exhibit direct anatomical connectivity with projection and association fibers traversing periventricular zones adjacent to the lateral ventricles. White matter tracts are recognized as fundamental structural substrates for cortical folding; however, while fiber bundle tension persists within the folded cortex, it does not directly drive the folding process itself (35). To further elucidate these relationships, we extracted specific white matter components associated with the DTI-ALPS index and analyzed their connectivity patterns with corresponding cortical regions. Interestingly, sulcal depth showed a mild positive correlation with MD in several DTI-ALPS-related fiber tracts. Given that elevated MD typically reflects reduced microstructural integrity or alterations in tissue organization, this finding suggests that regions with deeper sulci may have small but statistically significant correlations, with white matter exhibiting relatively higher diffusivity, which is contrary to our initial hypothesis. We speculate that deeper sulci, while providing a larger interface for CSF–interstitial fluid exchange, may concurrently exert mechanical stress on the underlying white matter, leading to less compact axonal packing or expansion of the extracellular space and thereby elevating MD. Furthermore, it should be noted that the partial correlation coefficients of the results above ranged from *r* = 0.117 to *r* = 0.224. Although these effects reached statistical significance in this large sample (*N* = 815), their magnitudes are small, and their biological relevance should be interpreted with caution. In a neurologically intact young cohort, even subtle associations may reflect meaningful variation in glymphatic function, but they are unlikely to represent robust individual-level predictors. Replication and mechanistic validation are needed to determine whether these modest relationships hold clinical or physiological significance.

Notably, although the DTI-ALPS index was computed as a bilateral average, the cortical regions and white matter tracts that showed significant associations with it were predominantly located in the left hemisphere. This pronounced left lateralization may reflect hemispheric asymmetry of the glymphatic system or white matter microstructure in right-handed individuals. Emerging evidence indicates that the relationships between the DTI-ALPS index, handedness, and language function exhibit lateralized patterns and suggests that hemispheric differences in brain function, together with certain methodological factors, may jointly contribute to this phenomenon (38). Future studies that compute hemisphere-specific DTI-ALPS index or systematically include left-handed participants will help to further elucidate the underlying biological asymmetry.

Several limitations of the present study should be acknowledged, namely: (1) The DTI-ALPS index is not fully equivalent to glymphatic function; rather, it primarily reflects local glymphatic activity through the perivenous fluid dynamics surrounding the medullary veins adjacent to the lateral ventricles. (2) Some of the observed correlations between cortical measures and white matter tracts in this study were contrary to our initial hypotheses. In particular, the fiber tracts that exhibited negative correlations with the DTI-ALPS index in the FA-based correlational tractography lack a sufficiently reasonable explanation. (3) This study was conducted exclusively in a healthy young population, and the findings have not been validated in aging or cognitively impaired populations. (4) The present study did not include an investigation of brain function. Future studies incorporating more refined brain atlases (e.g., Multimodal White Matter Atlas) (39), functional MRI measures (e.g., BOLD-CSF coupling), and relevant clinical scales will be of great importance to establish the relationships among glymphatic function, white matter fiber integrity, and brain function. (5) Because the tract–region pairs analyzed were preselected from significant associations identified in the same sample, the reported effect sizes may be inflated and should be interpreted with caution.

This study represents a large-scale investigation examining associations between the DTI-ALPS index and both white matter connectivity and cortical folding morphology. Our study provides a new direction for interpreting changes in the DTI-ALPS index in future research.

## Data Availability

Publicly available datasets were analyzed in this study. This data can be found here: https://openneuro.org/datasets/ds003097 AOMIC-ID1000.
