# Supplementary material for: Neuroanatomical substrates of perivascular space index: a morphometric and tractography study
Source: Front Psychiatry. 2026 Jun 19;17:1848053. doi: 10.3389/fpsyt.2026.1848053 (PMC13329724; doi:10.3389/fpsyt.2026.1848053)
Supplement: Supplementary file 1 [file Table1.docx]

eTable 1 Fiber composition and morphological parameters were associated with higher DTI-ALPS index and FA values

| **Tract Name** | | number of tracts | | mean length(mm) | | span(mm) | | curl | | elongation | | total volume(mm^3) | | 1st quarter volume(mm^3) | | 2nd and 3rd quarter volume(mm^3) | 4th quarter volume(mm^3) | | total surface area(mm^2) | |
| --- | --- | --- | --- | --- | --- | --- | --- | --- | --- | --- | --- | --- | --- | --- | --- | --- | --- | --- | --- | --- |
| **Corpus_Callosum_Body** | | 7446 | | 49.901196 | | 20.896318 | | 2.388038 | | 2.606959 | | 14360 | | 2485 | | 9163 | 2712 | | 24700 | |
| **Corpus_Callosum_Tapetum** | | 2743 | | 50.183079 | | 20.406448 | | 2.459177 | | 4.346462 | | 5254 | | 1036 | | 3163 | 1055 | | 11464 | |
| **Fornix_L** | | 1499 | | 46.660793 | | 19.310286 | | 2.41637 | | 4.524889 | | 3897 | | 871 | | 2183 | 843 | | 9552 | |
| **Fornix_R** | | 841 | | 46.194359 | | 19.332317 | | 2.389489 | | 4.506039 | | 3813 | | 627 | | 2314 | 872 | | 9249 | |
| **Thalamic_Radiation_Anterior_R** | | 505 | | 48.29874 | | 16.481022 | | 2.930567 | | 5.663342 | | 2759 | | 560 | | 1522 | 677 | | 7512 | |
| **Corpus_Callosum_ForcepsMajor** | | 303 | | 43.717861 | | 18.503725 | | 2.362652 | | 6.3142 | | 1646 | | 53 | | 1428 | 165 | | 5354 | |
| **Corticospinal_Tract_L** | | 266 | | 44.520424 | | 20.969234 | | 2.123131 | | 7.558811 | | 1213 | | 225 | | 586 | 402 | | 3028 | |
| **Anterior_Commissure_Occipital** | | 136 | | 51.431496 | | 23.814053 | | 2.159712 | | 11.637245 | | 789 | | 142 | | 483 | 164 | | 2569 | |
| **Anterior_Commissure_Temporal** | | 75 | | 52.802658 | | 24.151524 | | 2.186307 | | 12.508534 | | 739 | | 162 | | 437 | 140 | | 2880 | |
| **Thalamic_Radiation_Posterior_R** | | 57 | | 57.619606 | | 9.619095 | | 5.990128 | | 12.101151 | | 1026 | | 232 | | 532 | 262 | | 3625 | |
| **Thalamic_Radiation_Anterior_L** | | 32 | | 50.009872 | | 15.012456 | | 3.331225 | | 9.881652 | | 1006 | | 170 | | 534 | 302 | | 4734 | |
| **Cingulum_Frontal_Parahippocampal_L** | | 17 | | 55.520863 | | 16.937187 | | 3.278045 | | 14.893369 | | 606 | | 201 | | 239 | 166 | | 2947 | |
| **Superior_Longitudinal_Fasciculus_III_R** | | 15 | | 42.265427 | | 19.903065 | | 2.123564 | | 14.874948 | | 268 | | 76 | | 128 | 64 | | 1257 | |
| **Corticopontine_Tract_Frontal_L** | | 5 | | 43.187447 | | 20.267544 | | 2.130867 | | 19.82295 | | 161 | | 44 | | 72 | 45 | | 973 | |
| **NonDecussating_Dentatorubrothalamic_Tract_L** | | 5 | | 48.418434 | | 18.146206 | | 2.66824 | | 19.036781 | | 246 | | 48 | | 149 | 49 | | 1423 | |
| **Cingulum_Frontal_Parietal_R** | | 4 | | 62.029892 | | 23.911629 | | 2.594131 | | 27.548504 | | 247 | | 53 | | 150 | 44 | | 1626 | |
| **Corticopontine_Tract_Parietal_L** | | 3 | | 42.692204 | | 20.203283 | | 2.113132 | | 20.38962 | | 147 | | 32 | | 76 | 39 | | 1117 | |
| **Cingulum_Parolfactory_L** | | 1 | | 48.06554 | | 20.206985 | | 2.37866 | | 39.821201 | | 55 | | 12 | | 28 | 15 | | 560 | |
| **Optic_Radiation_R** | | 1 | | 72.013634 | | 11.391515 | | 6.321691 | | 59.808117 | | 82 | | 20 | | 42 | 20 | | 867 | |
|  | |  | |  | |  | |  | |  | |  | |  | |  |  | |  | |
|  | |  | |  | |  | |  | |  | |  | |  | |  |  | |  | |
|  | |  | |  | |  | |  | |  | |  | |  | |  |  | |  | |
| **Tract Name** | total radius of end regions(mm) | | total area of end regions(mm^2) | | irregularity | | area of end region 1(mm^2) | | radius of end region 1(mm) | | volume of end branches 1 | | area of end region 2(mm^2) | | radius of end region 2(mm) | | | volume of end branches 2 | |  |
| **Corpus_Callosum_Body** | 19.812412 | | 4818 | | 8.231128 | | 2275 | | 10.01773 | | 2485 | | 2543 | | 9.794683 | | | 2712 | |  |
| **Corpus_Callosum_Tapetum** | 16.31377 | | 1874 | | 6.298071 | | 883 | | 8.278554 | | 1036 | | 991 | | 8.035216 | | | 1055 | |  |
| **Fornix_L** | 10.993717 | | 1064 | | 6.318996 | | 490 | | 5.870659 | | 871 | | 574 | | 5.123058 | | | 843 | |  |
| **Fornix_R** | 11.701401 | | 821 | | 6.216729 | | 461 | | 5.512563 | | 627 | | 360 | | 6.188838 | | | 872 | |  |
| **Thalamic_Radiation_Anterior_R** | 13.166647 | | 575 | | 5.805062 | | 334 | | 8.596352 | | 560 | | 241 | | 4.570296 | | | 677 | |  |
| **Corpus_Callosum_ForcepsMajor** | 27.890776 | | 309 | | 5.630268 | | 175 | | 16.227669 | | 53 | | 134 | | 11.663107 | | | 165 | |  |
| **Corticospinal_Tract_L** | 5.584585 | | 267 | | 3.675707 | | 137 | | 2.656379 | | 225 | | 130 | | 2.928206 | | | 402 | |  |
| **Anterior_Commissure_Occipital** | 14.339741 | | 183 | | 3.597544 | | 97 | | 6.981574 | | 142 | | 86 | | 7.358167 | | | 164 | |  |
| **Anterior_Commissure_Temporal** | 14.620374 | | 124 | | 4.112798 | | 58 | | 8.193626 | | 162 | | 66 | | 6.426747 | | | 140 | |  |
| **Thalamic_Radiation_Posterior_R** | 8.448458 | | 89 | | 4.205758 | | 39 | | 3.428997 | | 232 | | 50 | | 5.019461 | | | 262 | |  |
| **Thalamic_Radiation_Anterior_L** | 14.693007 | | 56 | | 5.95383 | | 30 | | 11.485332 | | 170 | | 26 | | 3.207674 | | | 302 | |  |
| **Cingulum_Frontal_Parahippocampal_L** | 14.381144 | | 27 | | 4.532218 | | 15 | | 8.986194 | | 201 | | 12 | | 5.39495 | | | 166 | |  |
| **Superior_Longitudinal_Fasciculus_III_R** | 3.615172 | | 24 | | 3.331734 | | 14 | | 2.248492 | | 76 | | 10 | | 1.36668 | | | 64 | |  |
| **Corticopontine_Tract_Frontal_L** | 2.576874 | | 10 | | 3.291669 | | 5 | | 1.336305 | | 44 | | 5 | | 1.240568 | | | 45 | |  |
| **NonDecussating_Dentatorubrothalamic_Tract_L** | 6.249063 | | 10 | | 3.67813 | | 5 | | 4.238381 | | 48 | | 5 | | 2.010682 | | | 49 | |  |
| **Cingulum_Frontal_Parietal_R** | 10.207741 | | 8 | | 3.705669 | | 4 | | 7.73081 | | 53 | | 4 | | 2.476931 | | | 44 | |  |
| **Corticopontine_Tract_Parietal_L** | 3.692892 | | 6 | | 3.977546 | | 3 | | 1.961886 | | 32 | | 3 | | 1.731006 | | | 39 | |  |
| **Cingulum_Parolfactory_L** | 0 | | 2 | | 3.07245 | | 1 | | 0 | | 12 | | 1 | | 0 | | | 15 | |  |
| **Optic_Radiation_R** | 0 | | 2 | | 3.182731 | | 1 | | 0 | | 20 | | 1 | | 0 | | | 20 | |  |

eTable 2

Fiber composition and morphological parameters showed negative correlations with the DTI-ALPS index and FA values

| **Tract Name** | **Arcuate_Fasciculus_R** | **Arcuate_Fasciculus_L** | **Superior_Longitudinal_Fasciculus_R** |
| --- | --- | --- | --- |
| number of tracts | 67 | 56 | 10 |
| mean length(mm) | 43.769569 | 41.43391 | 40.999443 |
| span(mm) | 21.196554 | 19.858549 | 19.956989 |
| curl | 2.064938 | 2.086452 | 2.05439 |
| elongation | 11.664891 | 10.507602 | 14.373457 |
| total volume(mm^3) | 484 | 506 | 262 |
| 1st quarter volume(mm^3) | 157 | 114 | 56 |
| 2nd and 3rd quarter volume(mm^3) | 234 | 240 | 139 |
| 4th quarter volume(mm^3) | 93 | 152 | 67 |
| total surface area(mm^2) | 1742 | 1799 | 1295 |
| total radius of end regions(mm) | 6.468199 | 3.417461 | 6.220254 |
| total area of end regions(mm^2) | 91 | 66 | 20 |
| irregularity | 3.376248 | 3.504879 | 3.524725 |
| area of end region 1(mm^2) | 37 | 34 | 10 |
| radius of end region 1(mm) | 3.15239 | 1.970883 | 2.768641 |
| volume of end branches 1 | 157 | 114 | 56 |
| area of end region 2(mm^2) | 54 | 32 | 10 |
| radius of end region 2(mm) | 3.315808 | 1.446578 | 3.451613 |
| volume of end branches 2 | 93 | 152 | 67 |

eTable 3 Fiber composition and morphological parameters were negatively correlated with the DTI-ALPS index and MD values

| **Tract Name** | | number of tracts | | mean length(mm) | | span(mm) | | curl | | elongation | | total volume(mm^3) | | 1st quarter volume(mm^3) | | 2nd and 3rd quarter volume(mm^3) | | 4th quarter volume(mm^3) | | |
| --- | --- | --- | --- | --- | --- | --- | --- | --- | --- | --- | --- | --- | --- | --- | --- | --- | --- | --- | --- | --- |
| **Corpus_Callosum_Body** | | 13466 | | 54.376427 | | 19.930487 | | 2.728304 | | 2.220701 | | 25606 | | 3677 | | 18354 | | 3575 | | |
| **Corpus_Callosum_Tapetum** | | 7804 | | 52.42482 | | 18.42625 | | 2.845116 | | 2.970446 | | 12825 | | 1016 | | 8696 | | 3113 | | |
| **Fornix_L** | | 6413 | | 52.247597 | | 20.647749 | | 2.530426 | | 3.730555 | | 8049 | | 1854 | | 4420 | | 1775 | | |
| **Arcuate_Fasciculus_L** | | 2694 | | 49.549038 | | 21.167713 | | 2.340784 | | 2.833626 | | 11899 | | 2390 | | 7682 | | 1827 | | |
| **Fornix_R** | | 2617 | | 48.267845 | | 20.344807 | | 2.37249 | | 3.761276 | | 6243 | | 1037 | | 3876 | | 1330 | | |
| **Thalamic_Radiation_Anterior_L** | | 2161 | | 50.185268 | | 16.681095 | | 3.008512 | | 3.698799 | | 7256 | | 1084 | | 4464 | | 1708 | | |
| **Thalamic_Radiation_Anterior_R** | | 1809 | | 47.943264 | | 16.556898 | | 2.895667 | | 3.910117 | | 5661 | | 1013 | | 3345 | | 1303 | | |
| **Corticospinal_Tract_L** | | 881 | | 47.950962 | | 21.776705 | | 2.201938 | | 3.830051 | | 5903 | | 714 | | 4004 | | 1185 | | |
| **Superior_Longitudinal_Fasciculus_III_L** | | 731 | | 47.593685 | | 20.571667 | | 2.313555 | | 4.615305 | | 3975 | | 852 | | 2174 | | 949 | | |
| **Corticostriatal_Tract_Superior_L** | | 695 | | 46.858932 | | 21.735847 | | 2.155837 | | 3.882493 | | 5361 | | 796 | | 3556 | | 1009 | | |
| **Cingulum_Frontal_Parahippocampal_L** | | 586 | | 55.955372 | | 18.881046 | | 2.963574 | | 5.797406 | | 4094 | | 1047 | | 2471 | | 576 | | |
| **Superior_Longitudinal_Fasciculus_II_L** | | 456 | | 45.193947 | | 19.222071 | | 2.351149 | | 4.556467 | | 3492 | | 852 | | 2125 | | 515 | | |
| **NonDecussating_Dentatorubrothalamic_Tract_L** | | 401 | | 46.18618 | | 21.036728 | | 2.195502 | | 4.25895 | | 4266 | | 530 | | 2979 | | 757 | | |
| **Thalamic_Radiation_Posterior_R** | | 369 | | 49.471504 | | 13.54903 | | 3.651295 | | 5.650869 | | 2978 | | 472 | | 2131 | | 375 | | |
| **Corpus_Callosum_ForcepsMajor** | | 344 | | 44.80386 | | 19.653765 | | 2.279658 | | 5.494272 | | 2340 | | 138 | | 1249 | | 953 | | |
| **Corticopontine_Tract_Parietal_L** | | 238 | | 46.233383 | | 20.061312 | | 2.304604 | | 3.969861 | | 4925 | | 634 | | 3611 | | 680 | | |
| **Thalamic_Radiation_Posterior_L** | | 213 | | 46.431477 | | 22.084566 | | 2.10244 | | 5.542791 | | 2559 | | 476 | | 1487 | | 596 | | |
| **Medial_Lemniscus_L** | | 172 | | 46.199284 | | 18.960457 | | 2.436612 | | 4.658268 | | 3569 | | 268 | | 2679 | | 622 | | |
| **Cingulum_Superior_Longitudinal_Fasciculus_I_L** | | 145 | | 49.553658 | | 20.647139 | | 2.400026 | | 6.771886 | | 2084 | | 439 | | 1295 | | 350 | | |
| **Corticostriatal_Tract_Posterior_L** | | 109 | | 46.317459 | | 21.912533 | | 2.113743 | | 5.499867 | | 2580 | | 381 | | 1693 | | 506 | | |
| **Parietal_Aslant_Tract_L** | | 96 | | 43.004765 | | 18.189186 | | 2.364304 | | 7.669332 | | 1062 | | 241 | | 430 | | 391 | | |
| **Cingulum_Parolfactory_L** | | 72 | | 49.260574 | | 19.988485 | | 2.464448 | | 9.733238 | | 991 | | 128 | | 494 | | 369 | | |
| **Frontal_Aslant_Tract_L** | | 62 | | 48.161812 | | 20.809917 | | 2.314368 | | 8.470049 | | 1223 | | 207 | | 676 | | 340 | | |
| **Extreme_Capsule_L** | | 41 | | 54.596203 | | 22.094967 | | 2.470979 | | 10.697001 | | 1117 | | 215 | | 719 | | 183 | | |
| **NonDecussating_Dentatorubrothalamic_Tract_R** | | 41 | | 48.24638 | | 18.000212 | | 2.680323 | | 8.410249 | | 1247 | | 274 | | 711 | | 262 | | |
| **Corticopontine_Tract_Parietal_R** | | 37 | | 43.956242 | | 19.875593 | | 2.211569 | | 8.279791 | | 973 | | 182 | | 653 | | 138 | | |
| **Corticopontine_Tract_Frontal_L** | | 34 | | 43.006683 | | 19.008846 | | 2.262456 | | 10.070674 | | 616 | | 111 | | 369 | | 136 | | |
| **Corticostriatal_Tract_Posterior_R** | | 31 | | 43.691525 | | 21.120476 | | 2.068681 | | 9.45987 | | 732 | | 138 | | 371 | | 223 | | |
| **Medial_Lemniscus_R** | | 28 | | 46.145267 | | 20.480022 | | 2.253185 | | 8.71543 | | 1016 | | 115 | | 708 | | 193 | | |
| **Cingulum_Frontal_Parietal_R** | | 20 | | 47.204647 | | 18.405107 | | 2.564758 | | 11.442143 | | 631 | | 141 | | 315 | | 175 | | |
| **Middle_Longitudinal_Fasciculus_L** | | 18 | | 45.218712 | | 20.407143 | | 2.215828 | | 10.356854 | | 677 | | 97 | | 450 | | 130 | | |
| **Cingulum_Parahippocampal_Parietal_R** | | 17 | | 42.475319 | | 18.148344 | | 2.340451 | | 13.403788 | | 335 | | 88 | | 162 | | 85 | | |
| **Cingulum_Parolfactory_R** | | 12 | | 45.490833 | | 18.285666 | | 2.487787 | | 13.412504 | | 411 | | 67 | | 231 | | 113 | | |
| **Dentatorubrothalamic_Tract_lr** | | 11 | | 48.197647 | | 21.842592 | | 2.20659 | | 18.216326 | | 265 | | 42 | | 159 | | 64 | | |
| **Corticobulbar_Tract_R** | | 9 | | 46.232304 | | 17.558599 | | 2.633029 | | 15.220918 | | 335 | | 85 | | 159 | | 91 | | |
| **Optic_Radiation_R** | | 8 | | 41.503448 | | 18.644066 | | 2.226094 | | 13.819666 | | 294 | | 44 | | 173 | | 77 | | |
| **Thalamic_Radiation_Anterior_R** | | 4 | | 41.978279 | | 17.266245 | | 2.431234 | | 19.115396 | | 159 | | 36 | | 79 | | 44 | | |
| **Cingulum_Frontal_Parahippocampal_R** | | 3 | | 76.004158 | | 23.710775 | | 3.205469 | | 35.605457 | | 272 | | 15 | | 195 | | 62 | | |
| **Corticostriatal_Tract_Superior_R** | | 3 | | 43.970097 | | 19.745707 | | 2.226818 | | 21.097715 | | 150 | | 33 | | 91 | | 26 | | |
| **Hippocampus_Alveus_L** | | 2 | | 41.006855 | | 17.981443 | | 2.28051 | | 25.241791 | | 85 | | 23 | | 37 | | 25 | | |
| **Corticobulbar_Tract_L** | | 2 | | 46.047173 | | 19.46563 | | 2.365563 | | 31.557608 | | 77 | | 19 | | 36 | | 22 | | |
| **Cingulum_Frontal_Parietal_L** | | 1 | | 87.987717 | | 34.739685 | | 2.532773 | | 69.425102 | | 111 | | 27 | | 55 | | 29 | | |
| **Cingulum_Parahippocampal_Parietal_L** | | 1 | | 44.006554 | | 17.997097 | | 2.445203 | | 34.885075 | | 55 | | 13 | | 28 | | 14 | | |
| **Inferior_Fronto_Occipital_Fasciculus_L** | | 1 | | 41.99683 | | 20.700195 | | 2.028813 | | 32.522835 | | 55 | | 13 | | 25 | | 17 | | |
| **Inferior_Longitudinal_Fasciculus_L** | | 1 | | 42.014858 | | 20.656935 | | 2.033935 | | 35.204678 | | 47 | | 9 | | 23 | | 15 | | |
| **Optic_Radiation_L** | | 1 | | 40.022018 | | 17.354219 | | 2.306184 | | 30.821623 | | 53 | | 16 | | 25 | | 12 | | |
| **Corticopontine_Tract_Occipital_L** | | 1 | | 41.978207 | | 20.386955 | | 2.059072 | | 34.433624 | | 49 | | 13 | | 22 | | 14 | | |
|  | |  | |  | |  | |  | |  | |  | |  | |  | |  | | |
|  | |  | |  | |  | |  | |  | |  | |  | |  | |  | | |
| **Tract Name** | total surface area(mm^2) | | total radius of end regions(mm) | | total area of end regions(mm^2) | | irregularity | | area of end region 1(mm^2) | | radius of end region 1(mm) | | volume of end branches 1 | | area of end region 2(mm^2) | | radius of end region 2(mm) | | volume of end branches 2 |  |
| **Corpus_Callosum_Body** | 46630 | | 26.611567 | | 8399 | | 11.147675 | | 4581 | | 14.303091 | | 3677 | | 3818 | | 12.308475 | | 3575 |  |
| **Corpus_Callosum_Tapetum** | 27292 | | 22.74008 | | 4109 | | 9.389301 | | 2180 | | 10.59433 | | 1016 | | 1929 | | 12.14575 | | 3113 |  |
| **Fornix_L** | 17006 | | 15.88552 | | 2957 | | 7.397639 | | 1634 | | 9.164664 | | 1854 | | 1323 | | 6.720856 | | 1775 |  |
| **Arcuate_Fasciculus_L** | 22674 | | 20.068047 | | 2709 | | 8.330103 | | 1351 | | 9.816399 | | 2390 | | 1358 | | 10.251647 | | 1827 |  |
| **Fornix_R** | 13418 | | 14.180173 | | 1927 | | 6.895365 | | 957 | | 7.135934 | | 1037 | | 970 | | 7.044239 | | 1330 |  |
| **Thalamic_Radiation_Anterior_L** | 17933 | | 16.493004 | | 1748 | | 8.383228 | | 1063 | | 10.233633 | | 1084 | | 685 | | 6.25937 | | 1708 |  |
| **Thalamic_Radiation_Anterior_R** | 13131 | | 14.58231 | | 1448 | | 7.110211 | | 906 | | 8.464477 | | 1013 | | 542 | | 6.117833 | | 1303 |  |
| **Corticospinal_Tract_L** | 14962 | | 18.186602 | | 1075 | | 7.933223 | | 611 | | 11.615522 | | 714 | | 464 | | 6.571079 | | 1185 |  |
| **Superior_Longitudinal_Fasciculus_III_L** | 8713 | | 9.915039 | | 776 | | 5.650926 | | 444 | | 5.228209 | | 852 | | 332 | | 4.686831 | | 949 |  |
| **Corticostriatal_Tract_Superior_L** | 15717 | | 15.047112 | | 800 | | 8.845974 | | 437 | | 9.756136 | | 796 | | 363 | | 5.290977 | | 1009 |  |
| **Cingulum_Frontal_Parahippocampal_L** | 11080 | | 16.152653 | | 639 | | 6.530404 | | 381 | | 9.874199 | | 1047 | | 258 | | 6.278454 | | 576 |  |
| **Superior_Longitudinal_Fasciculus_II_L** | 10283 | | 13.287954 | | 618 | | 7.301929 | | 284 | | 6.532604 | | 852 | | 334 | | 6.755351 | | 515 |  |
| **NonDecussating_Dentatorubrothalamic_Tract_L** | 12586 | | 17.749176 | | 550 | | 7.998643 | | 296 | | 13.163837 | | 530 | | 254 | | 4.58534 | | 757 |  |
| **Thalamic_Radiation_Posterior_R** | 8664 | | 27.23329 | | 408 | | 6.367568 | | 208 | | 16.646568 | | 472 | | 200 | | 10.586721 | | 375 |  |
| **Corpus_Callosum_ForcepsMajor** | 10034 | | 23.198915 | | 362 | | 8.741854 | | 217 | | 15.751785 | | 138 | | 145 | | 7.44713 | | 953 |  |
| **Corticopontine_Tract_Parietal_L** | 17123 | | 27.245552 | | 387 | | 10.122643 | | 206 | | 16.041164 | | 634 | | 181 | | 11.204388 | | 680 |  |
| **Thalamic_Radiation_Posterior_L** | 7079 | | 11.095934 | | 331 | | 5.793294 | | 166 | | 5.553874 | | 476 | | 165 | | 5.54206 | | 596 |  |
| **Medial_Lemniscus_L** | 11853 | | 26.879351 | | 287 | | 8.234409 | | 140 | | 18.713226 | | 268 | | 147 | | 8.166124 | | 622 |  |
| **Cingulum_Superior_Longitudinal_Fasciculus_I_L** | 7469 | | 13.477623 | | 191 | | 6.556481 | | 93 | | 8.613205 | | 439 | | 98 | | 4.864418 | | 350 |  |
| **Corticostriatal_Tract_Posterior_L** | 9165 | | 16.418827 | | 198 | | 7.479032 | | 104 | | 9.677953 | | 381 | | 94 | | 6.740873 | | 506 |  |
| **Parietal_Aslant_Tract_L** | 3456 | | 4.894975 | | 129 | | 4.561926 | | 77 | | 2.52098 | | 241 | | 52 | | 2.373994 | | 391 |  |
| **Cingulum_Parolfactory_L** | 3710 | | 7.336436 | | 92 | | 4.736772 | | 58 | | 4.197338 | | 128 | | 34 | | 3.139099 | | 369 |  |
| **Frontal_Aslant_Tract_L** | 4976 | | 9.321497 | | 108 | | 5.783766 | | 53 | | 4.137751 | | 207 | | 55 | | 5.183746 | | 340 |  |
| **Extreme_Capsule_L** | 4707 | | 12.412193 | | 75 | | 5.376894 | | 36 | | 6.708042 | | 215 | | 39 | | 5.704151 | | 183 |  |
| **NonDecussating_Dentatorubrothalamic_Tract_R** | 5272 | | 11.095049 | | 77 | | 6.063241 | | 40 | | 8.664927 | | 274 | | 37 | | 2.430122 | | 262 |  |
| **Corticopontine_Tract_Parietal_R** | 4667 | | 19.900665 | | 66 | | 6.365994 | | 31 | | 9.951729 | | 182 | | 35 | | 9.948936 | | 138 |  |
| **Corticopontine_Tract_Frontal_L** | 2500 | | 8.670635 | | 54 | | 4.332881 | | 27 | | 5.109633 | | 111 | | 27 | | 3.561002 | | 136 |  |
| **Corticostriatal_Tract_Posterior_R** | 3053 | | 5.110443 | | 50 | | 4.815792 | | 26 | | 3.123205 | | 138 | | 24 | | 1.987238 | | 223 |  |
| **Medial_Lemniscus_R** | 4845 | | 19.153738 | | 54 | | 6.312166 | | 28 | | 8.901341 | | 115 | | 26 | | 10.252397 | | 193 |  |
| **Cingulum_Frontal_Parietal_R** | 3198 | | 6.772293 | | 33 | | 5.227168 | | 19 | | 3.160057 | | 141 | | 14 | | 3.612236 | | 175 |  |
| **Middle_Longitudinal_Fasciculus_L** | 4545 | | 22.474842 | | 35 | | 7.327832 | | 17 | | 12.25668 | | 97 | | 18 | | 10.218163 | | 130 |  |
| **Cingulum_Parahippocampal_Parietal_R** | 1493 | | 3.820538 | | 31 | | 3.530727 | | 17 | | 2.313823 | | 88 | | 14 | | 1.506714 | | 85 |  |
| **Cingulum_Parolfactory_R** | 2465 | | 9.065903 | | 22 | | 5.085447 | | 12 | | 2.761326 | | 67 | | 10 | | 6.304577 | | 113 |  |
| **Dentatorubrothalamic_Tract_lr** | 1368 | | 10.002928 | | 20 | | 3.414643 | | 11 | | 5.91785 | | 42 | | 9 | | 4.085077 | | 64 |  |
| **Corticobulbar_Tract_R** | 1827 | | 4.897353 | | 17 | | 4.141316 | | 8 | | 2.961141 | | 85 | | 9 | | 1.936212 | | 91 |  |
| **Optic_Radiation_R** | 1964 | | 11.063978 | | 15 | | 5.015575 | | 8 | | 3.031801 | | 44 | | 7 | | 8.032177 | | 77 |  |
| **Thalamic_Radiation_Anterior_R** | 1017 | | 3.237157 | | 8 | | 3.5116 | | 4 | | 1.583297 | | 36 | | 4 | | 1.653861 | | 44 |  |
| **Cingulum_Frontal_Parahippocampal_R** | 2711 | | 34.666069 | | 6 | | 5.318895 | | 3 | | 19.495985 | | 15 | | 3 | | 15.170082 | | 62 |  |
| **Corticostriatal_Tract_Superior_R** | 1487 | | 20.283686 | | 5 | | 5.165135 | | 3 | | 8.853872 | | 33 | | 2 | | 11.429813 | | 26 |  |
| **Hippocampus_Alveus_L** | 688 | | 1.368856 | | 4 | | 3.287349 | | 2 | | 0.838526 | | 23 | | 2 | | 0.53033 | | 25 |  |
| **Corticobulbar_Tract_L** | 672 | | 1.179849 | | 4 | | 3.183593 | | 2 | | 0.649519 | | 19 | | 2 | | 0.53033 | | 22 |  |
| **Cingulum_Frontal_Parietal_L** | 1116 | | 0 | | 2 | | 3.185567 | | 1 | | 0 | | 27 | | 1 | | 0 | | 29 |  |
| **Cingulum_Parahippocampal_Parietal_L** | 595 | | 0 | | 2 | | 3.411709 | | 1 | | 0 | | 13 | | 1 | | 0 | | 14 |  |
| **Inferior_Fronto_Occipital_Fasciculus_L** | 550 | | 0 | | 2 | | 3.228258 | | 1 | | 0 | | 13 | | 1 | | 0 | | 17 |  |
| **Inferior_Longitudinal_Fasciculus_L** | 512 | | 0 | | 2 | | 3.250234 | | 1 | | 0 | | 9 | | 1 | | 0 | | 15 |  |
| **Optic_Radiation_L** | 550 | | 0 | | 2 | | 3.368762 | | 1 | | 0 | | 16 | | 1 | | 0 | | 12 |  |
| **Corticopontine_Tract_Occipital_L** | 545 | | 0 | | 2 | | 3.389859 | | 1 | | 0 | | 13 | | 1 | | 0 | | 14 |  |
